# Characterization of Hand Clenching in Human Sensorimotor Cortex Using High-, and Ultra-High Frequency Band Modulations of Electrocorticogram

**DOI:** 10.3389/fnins.2018.00110

**Published:** 2018-02-27

**Authors:** Tianxiao Jiang, Su Liu, Giuseppe Pellizzer, Aydin Aydoseli, Sacit Karamursel, Pulat A. Sabanci, Altay Sencer, Candan Gurses, Nuri F. Ince

**Affiliations:** ^1^Clinical Neural Engineering Lab, Department of Biomedical Engineering, University of Houston, Houston, TX, United States; ^2^Research Service, Minneapolis VA Health Care System, Departments of Neurology and Neuroscience, University of Minnesota, Minnesota, MN, United States; ^3^Department of Neurosurgery, Istanbul Faculty of Medicine, Istanbul University, Istanbul, Turkey; ^4^Department of Physiology, Faculty of Medicine, Istinye University, Istanbul, Turkey; ^5^Department of Neurology, Istanbul Faculty of Medicine, Istanbul University, Istanbul, Turkey

**Keywords:** hybrid high-density grid, ECoG, ultra-high frequency band, hand movement, sensorimotor, postcentral shift

## Abstract

Functional mapping of eloquent cortex before the resection of a tumor is a critical procedure for optimizing survival and quality of life. In order to locate the hand area of the motor cortex in two patients with low-grade gliomas (LGG), we recorded electrocorticogram (ECoG) from a 113 channel hybrid high-density grid (64 large contacts with diameter of 2.7 mm and 49 small contacts with diameter of 1 mm) while they executed hand clenching movements. We investigated the spatio-spectral characteristics of the neural oscillatory activity and observed that, in both patients, the hand movements were consistently associated with a wide spread power decrease in the low frequency band (LFB: 8–32 Hz) and a more localized power increase in the high frequency band (HFB: 60–280 Hz) within the sensorimotor region. Importantly, we observed significant power increase in the ultra-high frequency band (UFB: 300–800 Hz) during hand movements of both patients within a restricted cortical region close to the central sulcus, and the motor cortical “hand knob.” Among all frequency bands we studied, the UFB modulations were closest to the central sulcus and direct cortical stimulation (DCS) positive site. Both HFB and UFB modulations exhibited different timing characteristics at different locations. Power increase in HFB and UFB starting before movement onset was observed mostly at the anterior part of the activated cortical region. In addition, the spatial patterns in HFB and UFB indicated a probable postcentral shift of the hand motor function in one of the patients. We also compared the task related subband modulations captured by the small and large contacts in our hybrid grid. We did not find any significant difference in terms of band power changes. This study shows initial evidence that event-driven neural oscillatory activity recorded from ECoG can reach up to 800 Hz. The spatial distribution of UFB oscillations was found to be more focalized and closer to the central sulcus compared to LFB and HFB. More studies are needed to characterize further the functional significance of UFB relative to LFB and HFB.

## 1. Introduction

Functional brain mapping is essential to improve the outcome of the neurosurgery by maximizing the excision while minimizing neurological deficits (Sanai and Berger, 2008; Chang et al., 2011). However, the mapping of the eloquent areas of the brain is a complex procedure due to the large variability of functional cortical organization between individuals (Brett et al., 2002; Farrell et al., 2007), as well as to the functional reorganization caused by brain injury, such as brain tumor (Dancause et al., 2005; Kong et al., 2016). As of today, direct cortical stimulation mapping (DCS) is still deemed as the gold standard in clinical practice. By directly injecting current to the cortical surface, DCS can either induce involuntary movement or suppress voluntary movement depending on the functional region (Brunner et al., 2009). However, some drawbacks of DCS are that it is time-consuming to adjust stimulation parameters, and test successively stimulation sites, and that it may induce spread of cortical activation that elicit seizures.

In the past decade, there has been a growing interest in the use of electrocorticogram (ECoG) to map functional areas without delivering electrical current to the cortex. By placing an electrode grid directly onto the cortex, it becomes possible to record oscillatory activity of the cortical circuits with unparalleled temporal and spatial resolution, as well as high signal quality. Previous work has shown that sensorimotor activity is associated with sub-band modulations of neural oscillations in the form of event-related desynchronization (ERD) in the alpha (7–13 Hz) and beta (13–32 Hz) bands and in the form of event-related synchronization (ERS) in the gamma band ranging from 40 to 200 Hz (Pfurtscheller and Lopes da Silva, 1999; Miller et al., 2007a). Recently it has been found that cognitive tasks related ECoG power modulations existed in an even broader band (60–500 Hz) (Gaona et al., 2011). High frequency band modulations are thought to be related to local neuronal processing while low frequency band changes are thought to reflect cortico-cortical, and cortico-subcortical interactions (Su and Ojemann, 2013). Although the exact physiological mechanisms underlying different subband modulations are yet to be elucidated, studies comparing ECoG functional mapping with DCS results generally showed that ERD in alpha and beta band were widespread and had low spatial specificity (Crone et al., 1998a; Leuthardt et al., 2007; Vansteensel et al., 2013). These clinical studies suggested that gamma band ERS correlated better with DCS in terms of specificity and sensitivity (Crone et al., 1998a). In recent years, ERS in gamma band has been proposed and successfully used for real-time functional mapping applications as well as brain machine interfaces (BMI) (Schalk et al., 2008; Miller et al., 2009, 2010; Hochberg et al., 2012; Branco et al., 2017).

Previous ECoG based functional brain mapping studies generally utilized regular clinical grid electrodes with large inter-electrode distance (1 cm) and small number of channels (<64) (Crone et al., 1998b; Aoki et al., 1999; Sinai et al., 2005; Leuthardt et al., 2007, 2012; Miller et al., 2007a,b, 2012; Schalk et al., 2008; Vansteensel et al., 2013). The frequency band of interest investigated in these studies were generally limited to 250 Hz. In this study, in order to map the hand function on the cortex, we recorded 113-channel high-density ECoG at 2.4 kHz from two patients with LGG while they performed voluntary hand clenching movements. There is ample evidence that these hand movements are controlled as a unit through motor synergies, rather than by individual control of fingers (Mason et al., 2001; Santello et al., 2013; Leo et al., 2016). We computed ECoG derived functional mapping in typical frequency bands of LFB and HFB. We also found that a small number of channels were associated with significant power modulations in an ultra-high frequency band ranging from 300 to 800 Hz. To the best of our knowledge, this is the first report showing that ECoG spectral modulations recorded from the hand area of the motor cortex can reach up to 800 Hz. These ultra high frequency modulations were found to be focally localized adjacent to the central sulcus, and close to the “hand knob” of the motor cortex (Yousry et al., 1997), and the DCS positive site. Moreover, in one of the patients, our ECoG based mapping results suggested that there had been a cortical reorganization of the hand motor function posterior to the central sulcus.

## 2. Materials and methods

### 2.1. Patients

Two right-handed male patients (P1 and P2) who were scheduled for resection of LGG requiring a craniotomy over the left motor and somatosensory brain areas were included in the study. Both patients gave informed consent before their participation to the study in accordance with the Declaration of Helsinki. The study protocol was reviewed and approved by the Institutional Review Boards (IRB) of Istanbul University, and of the University of Houston.

Functional mapping using intracranial electrodes were required to guide the resective surgeries since the tumor in both patients was in proximity to the motor cortex. A customized 113-channel hybrid electrode grid (INC electrode, CorTec GmbH) was used to map the border between the tumor and eloquent areas. The grid was positioned in a way to cover the border of the tumor and extend toward the “hand knob” of the primary motor area (M1). These hybrid ECoG grids have twice the density of typical clinical ECoG grids, which generally have 1 cm spacing between contacts, therefore providing higher spatial resolution. Specifically, the grid has 64 large contacts (MP35N nickel-cobalt alloy of 2.7 mm diameter, spaced every 1 cm) interlaced with 49 small contacts (platinum-iridium alloy of 1 mm diameter, spaced every 1 cm) and embedded in medical grade silicon rubber substrate (Figure 3). The spacing between adjacent small and large contacts is around 7 mm. The overall dimension of the electrode grid was 86 × 80 × 0.4 mm.

**Figure 1:**
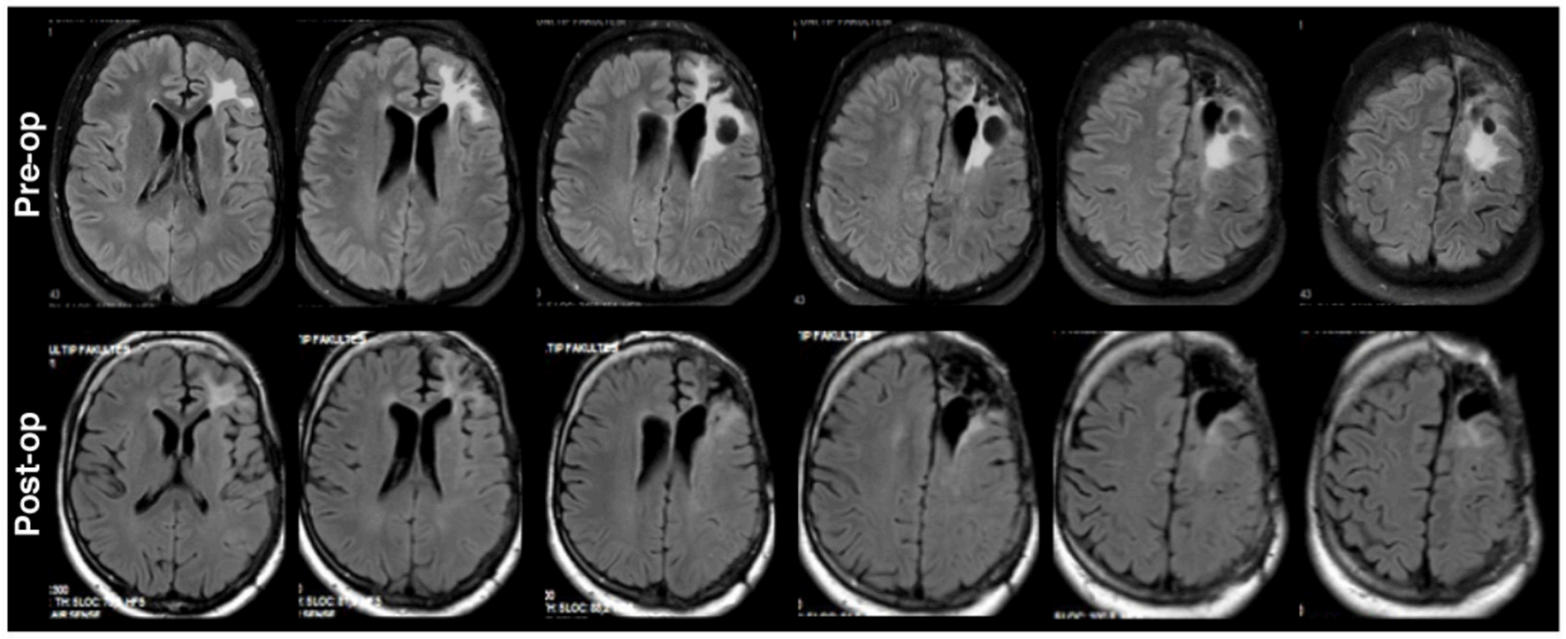
Axial fluid-attenuated inversion recovery (FLAIR) brain MRI for P1 shown in radiological convention. Preoperative left frontal hyperdensities **(upper)** around the previous operation tumor space were reduced in size in postoperative MRI **(lower)**.

The first patient (P1) was a 19 years old male who was initially diagnosed and operated for epilepsy at the age of 7. Histopathological investigations revealed a World Health Organization (WHO) grade II glioma (Louis et al., 2007). He received another operation 3 years prior to the current study. In this study, the patient was admitted to the clinic for a new onset of absence seizures which were resistant to antiepileptic drugs (oxcarbazepine 600 mg 3 × 1, levetiracetam 1,000 mg 3 × 1, topiramate 100 mg 3 × 1, lacosamide 100 mg 2 + 1 + 2). The preoperative and postoperative MRIs are provided in Figure 1. The head MRI indicated a recurrent left frontal tumor adjacent to the motor cortex. The patient had no functional deficit postoperatively. However, although reduced, the patient was not seizure free at follow up of 1 year according to a routine neurological examination under his antiepileptic medical therapy.

**Figure 2:**
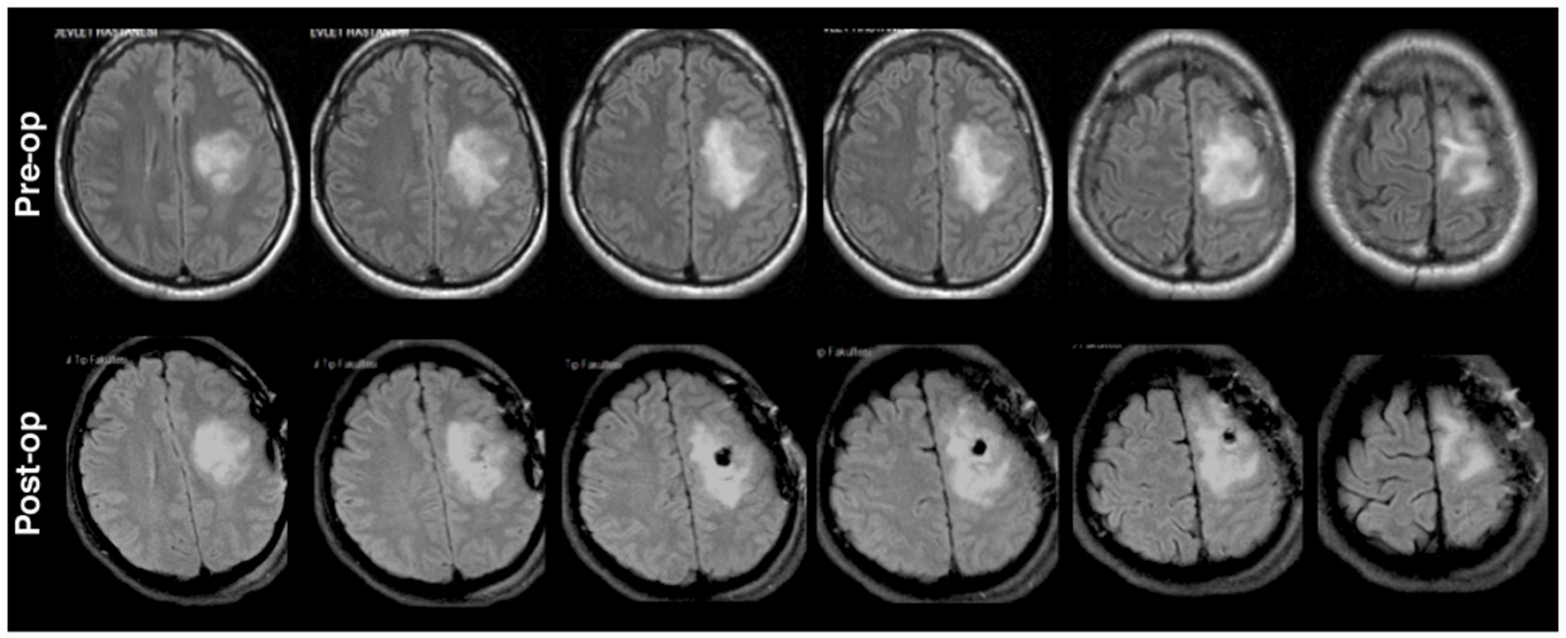
Preoperative **(upper)** and postoperative **(lower)** axial MRI FLAIR sequences for P2. Surgery was terminated with incomplete resection due to determination of right hand weakness.

The second patient (P2) was a 30 years old male who was admitted to the clinic due to right sided numbness affecting his hand for the previous 3 months. The head MRI (Figure 2) revealed a left posterior frontal cortico-subcortical tumor seated under the motor cortex. The determination of right hand weakness during surgery prompted its termination without further tumor resection (Figure 2). The patient had a postoperative right hand paresis that improved after 3 months. Pathological investigation revealed a WHO grade II glial tumor.

The clinical profiles of both patients and experiment information are shown in Table 1.

**Table 1:** Summary of subjects and experiments.

**ID**	**Case**	**Sex**	**Age**	**HN^a^**	**LGG Site**	**Trials**
P1	LGG, EP^b^	M^c^	19	Right	Left frontal	18
P2	LGG	M	30	Right	Left posterior frontal	30

a *Handedness*.

b *Epilepsy*.

c *Male*.

### 2.2. ECoG recordings and direct cortical stimulation

The ECoG recordings for patient P1 was carried out in the epilepsy monitoring unit (EMU) immediately after implantation of the ECoG grid for prolonged monitoring of seizure onset zone. For patient P2, ECoG recordings were performed in the operating room (OR) during awake-surgery to map and monitor the hand function throughout the resection. For both patients, ECoG data related to hand movements was obtained prior to DCS and surgical resection.

During the recordings, patients were asked to perform an alternate hand clenching and relax task. In each trial, subjects were instructed verbally to close the hand to a fist (i.e., hand clenching) and maintain this posture for around 2–3 s until instruction to relax. A resting period of at least 2 s was maintained between hand relaxation and the consecutive hand closing. ECoG, bipolar electrocardiogram (ECG), bipolar surface electromyogram (EMG) of the forearm flexor muscles, and hand movement data were recorded during the experiment with an in-house custom-made interface (Jiang et al., 2017b).

All biosignals including ECoG, bipolar ECG, and forearm EMG were recorded with a 256 channel bioamplifer gHIamp (g.tec medical engineering GmbH, Graz Austria) through an oversampling process at 2.4 kHz and 24 bit A/D resolution. To be more specific, the amplifier first digitized the signals at 614.4 kHz which is much higher than the required sampling frequency. Then, internally, the floating point digital signal processor (DSP) of the amplifier performed averaging of samples to increase the signal-to-noise ratio (SNR) and decimated the signal to the desired rate of 2.4 kHz.

**Figure 3:**
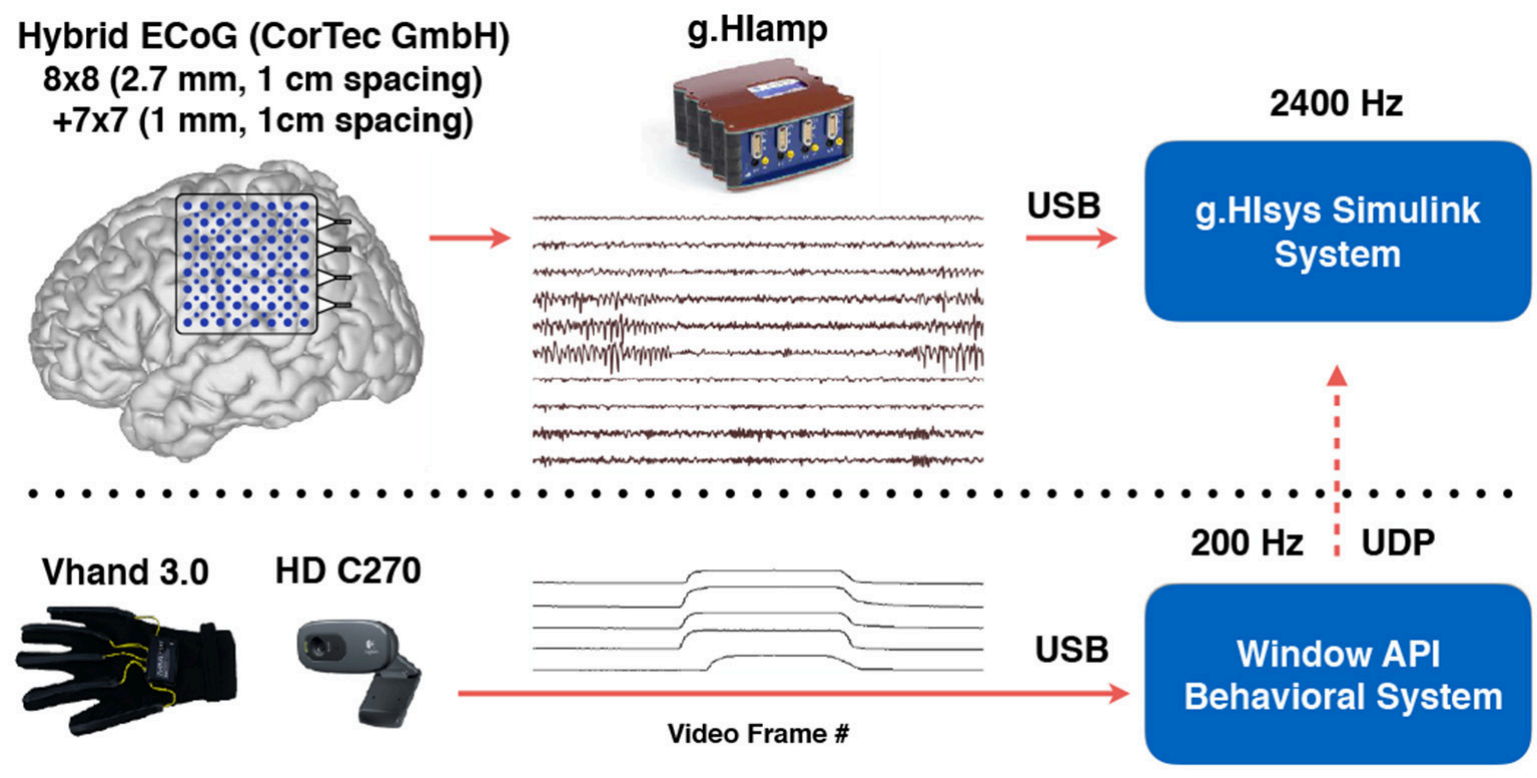
Recording setup to collect ECoG and sensor data simultaneously. The layout of the custom hybrid ECoG grid with 113 channels was shown on a template brain surface. Video and hand position data were captured by the behavioral system and sent to g.HIsys Simulink system via UDP to synchronize with neural data (Jiang et al., 2017b).

The signal acquisition and real-time visualization was executed with a customized Simulink model (Matlab R2014a, Mathworks, Inc) and gHIsys real-time signal processing library (g.tec medical engineering GmbH, Graz Austria). The hand movements of both patients were recorded with a dataglove (DG5 VHand 3.0) and a high-definition webcam (Logitech HD C270). The finger positions and video frame-timestamp were recorded at 200 Hz with custom in-house software that we developed in C++ running under Windows 7 OS and transmitted over Ethernet via User Datagram Protocol (UDP) at 100 Mbits/s to the Simulink model (Figure 3). Video frame timestamps and finger position data were upsampled to 2.4 kHz for synchronization of neural data with behavioral data. Detailed system specifications can be found in our earlier publication (Jiang et al., 2017b).

Mapping of the hand area using DCS was performed on both patients after the ECoG recordings. Stimulation was conducted between pair of contacts with a current amplitude ranging from 1 to 15 mA, pulse width of 200–300 μs, and duration of 0.2 s, according to the patient;s individual tolerance. However, since both patients suffered from tumor related epilepsy, and DCS sometimes produced after discharges, the first site that elicited hand movements was labeled as DCS positive site. No additional stimulation was performed after that due to the risk of inducing seizures.

### 2.3. Electrode localization and relative distance to central sulcus

**Figure 4:**
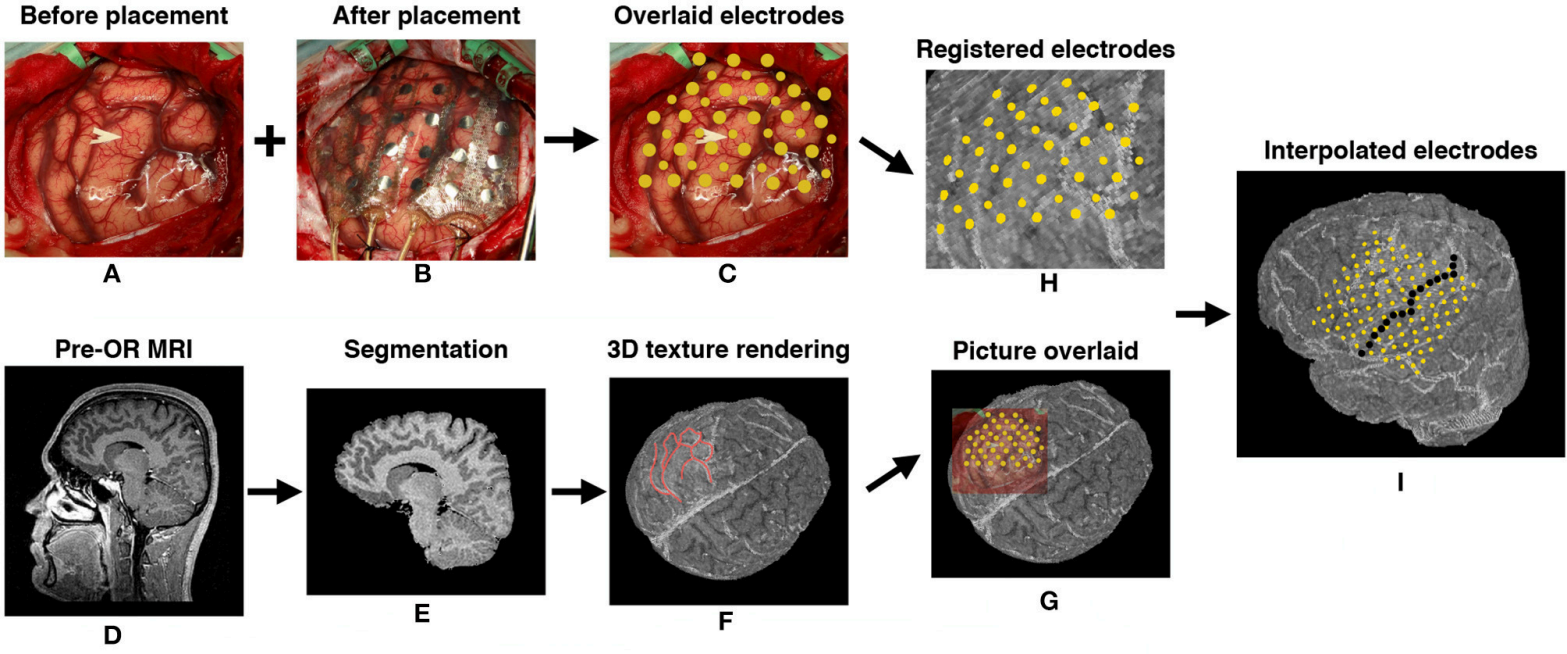
Intraoperative photo based electrode registration procedure for P2. **(A)** Cortex photo before electrode placement. **(B)** Photo taken from the same position after electrode placement. **(C)** Electrodes (disks in gold) manually segmented out and overlaid on the cortex. **(D)** Patient's preoperative MRI. **(E)** Gray and white matter segmentation. **(F)** 3D texture rendering of gray and white matter. Blood vessels were accentuated by red lines as landmarks. **(G)** Picture overlaid on 3D rendering by comparing the landmarks. **(H)** Electrodes registered on 3D rendering of the brain. **(I)** The side view of the cortex with registered and interpolated electrodes, central sulcus was delineated by a series of discrete black points.

Since the experiments were performed in the EMU for P1, postoperative CT after ECoG implantation and preoperative MRI were used to coregister the electrode positions. For P2, intraoperative photography and preoperative MRI were used instead for electrode localization as intraoperative CT was not available (Dalal et al., 2008; Gupta et al., 2014). For P1, CT+MRI coregistration and electrode segmentation were performed using Curry (version 7.0, Compumedics Neuroscan, Charlotte, NC, USA) by a trained neurologist. For P2, craniotomy photos were taken from the same position before and after the electrode placement. Each visible contact was manually segmented out and marked on the craniotomy picture taken before the electrode placement. Gray matter and white matter were automatically segmented out using SPM12 (Kiebel et al., 1997). The segmented volume was rendered in Matlab and visually compared with the craniotomy picture. Landmarks such as blood vessels, sulci and gyri were used to co-register the electrodes from the photo and the rendered MRI volume. The positions of contacts that were not visually exposed were iteratively interpolated from the neighboring contacts (Figure 4). The photo based electrode localizations procedure was also performed for P1 and the result was compared with the CT+MRI based method for an estimate of concordance of the two methods.

To compute the relative distance between the electrodes and the central sulcus, the central sulcus outline was delineated on the individual MRI by a series of discrete points (Figure 4I). The continuous curve of the central sulcus was approximated by consecutive linear segments. The relative distance from each contact to the central sulcus was defined as the minimal Euclidean distance to all the linear segments.

### 2.4. Preprocessing

**Figure 5:**
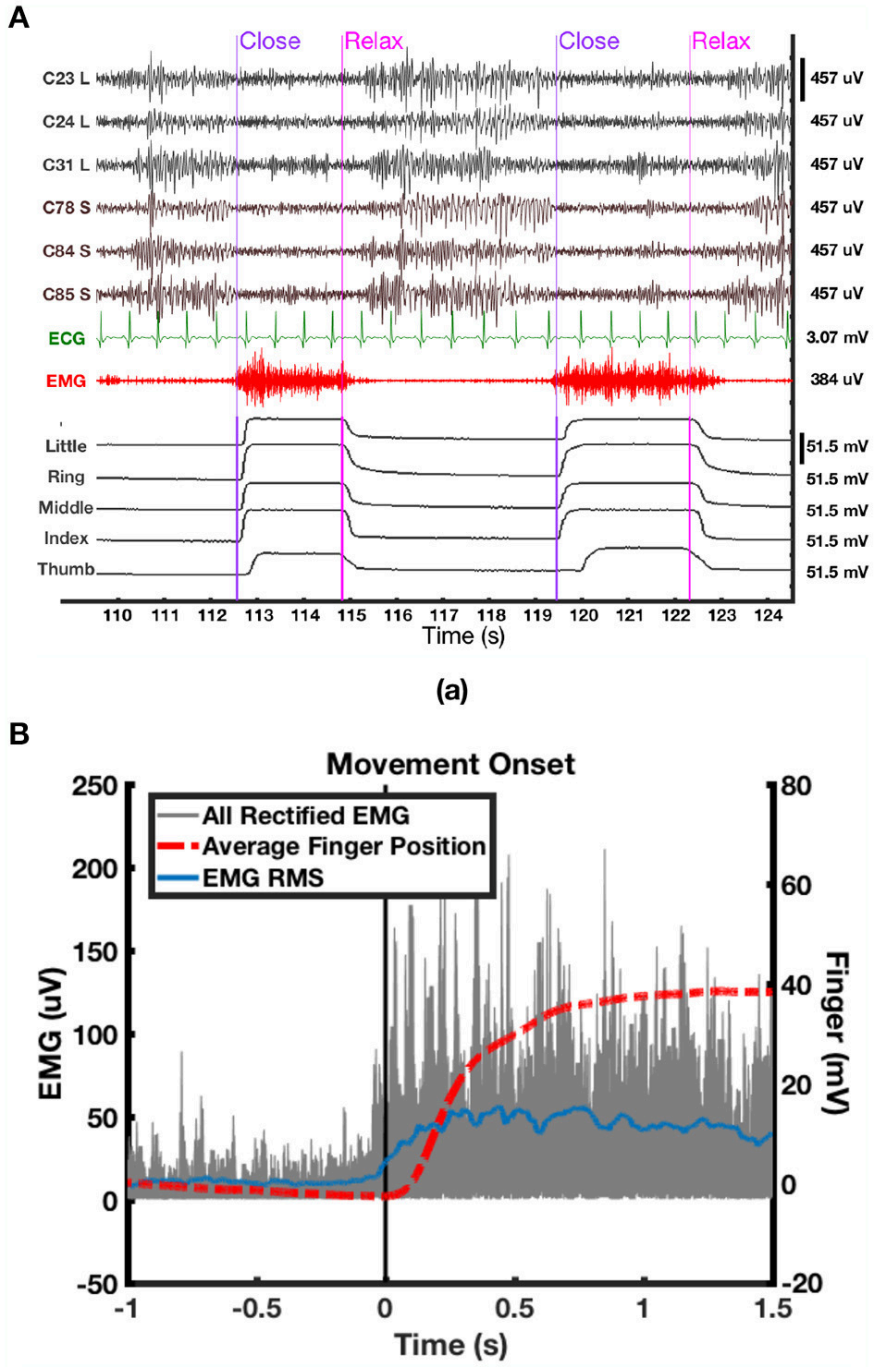
**(A)** Synchronized epoch of data of ECoG, ECG, EMG, and finger positions data of P2. ECoG were high-pass filtered at 3 Hz, whereas EMG was high-pass filtered at 50 Hz for visualization. **(B)** Overlay of rectified EMG (gray) from uncorrupted trials, average of all five finger positions from the dataglove (red) and root mean square (RMS) average of rectified EMG (blue).

All data were scrutinized in Matlab, and corrupted channels were excluded from further analysis. Incomplete and noisy trials were also excluded by visually checking the neural and behavioral data with synchronized video recordings offline. Second-order Butterworth IIR notch filters with 2 Hz stop band were applied to eliminate the effect of 50 Hz power line noise and its harmonics. Movement onset from hand relaxed to clenched was determined using the minimum acceleration criterion with constraints (MACC) method (Botzer, 2009) on the dataglove data. The earliest movement onset detected among all five digits was used as the onset of hand clenching. We found that the output of the dataglove was more reliable than EMG to determine movement onset partly due to artifacts and occasional bursts of EMG that were not associated with hand movements, as could be verified from the video. An epoch of ECoG data, uncorrupted EMG, synchronized finger positions and movement onsets automatically determined using MACC is shown in Figure 5A. For selected trials with uncorrupted EMG, the onset of EMG signal was found to be 50 ms prior to the onset of the finger positions changes measured from the dataglove data (Figure 5B). To account for this delay, movement onset determined from the dataglove signal was shifted 50 ms earlier in the following analyses.

### 2.5. Time-frequency and power spectral density analysis

For each channel, a time-frequency analysis was performed using short-time Fourier transform (STFT) on 3 s of ECoG data centered at each hand closing onset. Specifically, a 1,024-sample FFT was computed in each 1,024-sample Hanning window shifted with 90% overlap. Denoting movement onset as 0 s, each channel spectrogram (*S*_*C*_) was computed by averaging across trials (*S*_*A*_) and then normalized by the spectrum of the baseline period starting from −1.5 s and extending to −1 s (*S*_*R*_). The normalized spectrogram (*S*_*C*_) was transformed into dB scale:

(1) $$\begin{array}{r} S_{C}=10\times \log_{10}\frac{S_{A}}{S_{R}} \end{array}$$

The normalized time-frequency maps (*S*_*C*_) were visualized to investigate the ERD and ERS patterns in active channels of the electrode grid.

The power spectral densities of ECoG during baseline and hand close were estimated using Welch's method from 0.5 s of data segments. The baseline segment was the same as for the time-frequency analysis, while the hand movement segment was selected as −0.1 to +0.4 s to cover the movement initiation period. While the oversampling process executed by the amplifier provides exceptional SNR, the averaging step before decimation has a narrow band low-pass filtering effect where its passband response is not flat and has a droop. Consequently, the estimated power spectrum of ECoG does not have a visible flat noise floor and follows the magnitude response of the averaging filter which causes difficulties in the interpretation of the spectrum. In practice, in order to compensate the passband droop and obtain a flat passband response, an FIR filter is generally used after decimation (Lyons, 2004) with a magnitude response that is ideally an inverted version of the averaging filter passband response (Lyons, 2004). For this reason, we corrected the estimated spectrum with the inverted magnitude of the averaging filter in the passband.

Based on initial observations, the power spectral densities were estimated in two groups of channels for each patient: channels with ERS restricted in HFB and channels with ERS clearly extended to a higher frequency range.

Furthermore, we investigated three reactive frequency bands. The 8–32 Hz low frequency band (LFB); the 60–280 Hz high frequency band (HFB); and the 300–800 Hz ultra-high frequency band (UFB). The LFB was selected for its well-known movement-related ERD (Pfurtscheller and Lopes da Silva, 1999). The high frequency band (HFB) was selected to cover the high gamma activity where typically peri-movement ERS occurs (Miller et al., 2007a). In addition, we investigated an even higher frequency band at 300-800 Hz that manifested ERS during movements. We referred to this latter band as ultra-high frequency band (UFB) to distinguish it from the traditional high gamma range.

### 2.6. Spatial patterns of LFB, HFB, and UFB and relative distance to DCS(+) site

The movement-related spatial pattern of each frequency band from 0.1 s before movement onset (−0.1 s) to 0.4 s after it (+0.4 s) was obtained by computing the subband power ratio (*R*_*p*_) between movement (*P*_*m*_) and baseline (*P*_*b*_) of individual channels and expressed in dB scale:

(2) $$\begin{array}{r} R_{p}=10\times \log_{10}\frac{P_{m}}{P_{b}} \end{array}$$

The spatial matrices obtained from each channel's *R*_*p*_ in LFB, HFB, and UFB were interpolated by performing Delaunay triangulation (Lee and Schachter, 1980) on the registered electrode positions and visualized on the individual MRI rendering.

Channels were defined with significant ERD or ERS, when the change of power during hand clenching (*R*_*p*_) was significantly >25% relative to baseline. The statistical significance of ERD in LFB, and ERS in HFB and UFB was tested using a one-tailed Student's *t*-test with a significance threshold *p*-value of 0.05 and corrected for multiple comparison by false discovery rate (FDR) method at the level of 0.05 (Genovese et al., 2002). For ERD, the alternative hypothesis (*H*_1_) is *R*_*p*_ < 0.75 (−1.25 dB), while for ERS, *H*_1_ is *R*_*p*_ > 1.25 (+0.97 dB). The sample population of the *t*-tests for each patient and channel was comprised of all hand clenching trials (P1: 18, P2: 30).

We also defined two metrics to compare DCS results and spatial patterns of different frequency bands. The distance between the peak activated electrode position of each subband and the DCS(+) site across the grid was defined as *d*_*p*_. In addition, the distance between the activation map centroid for each subband and the DCS(+) site was defined as *d*_*c*_. The map centroid was defined as the weighted summation of significant channel positions by the activation magnitude *R*_*p*_ of the subband. Both distance metrics were calculated on the 2D plane of the grid.

### 2.7. Early vs. late ERS onset

The temporal characteristics of the ERS across channels were studied by categorizing them into two groups, the early ERS group and the late ERS group. The early ERS group was determined by a significant power increase in HFB or UFB range using data segments from −0.5 to 0 s where 0 s represents movement onset. While the late ERS group was determined by a significant power increase exclusively in the data segments from 0 to 0.5 s. In both cases, the baseline activity was selected from −1.5 to −1 s as before. The significances of HFB-ERS and UFB-ERS of each channel were tested using one-tailed Student's *t*-test (*p* = 0.05) with the null hypothesis that the power in each subband is equal to the baseline. To correct for multiple comparisons of channels, the Bonferroni correction was applied.

### 2.8. Small vs. large contact groups

The power spectral densities for small and large contacts during baseline were estimated using Welch's method and averaged across each contact group. The ECoG noise floor of each channel was estimated using the band power within 800–1,000 Hz. The signal-to-noise ratio (SNR) in each band was computed from the ratio of the band power to the estimated noise floor. Channels with significant ERS in HFB were selected to compute the SNR in HFB and UFB between small and large contacts during both baseline and movement periods. In addition, the magnitude of ERD and ERS captured between small and large contacts were also compared within the selected HFB-ERS channels. A two-tailed Student's *t*-test with a *p*-value of 0.05 was used for significance test.

## 3. Results

### 3.1. Power spectral density estimation and time-frequency analysis

**Figure 6:**
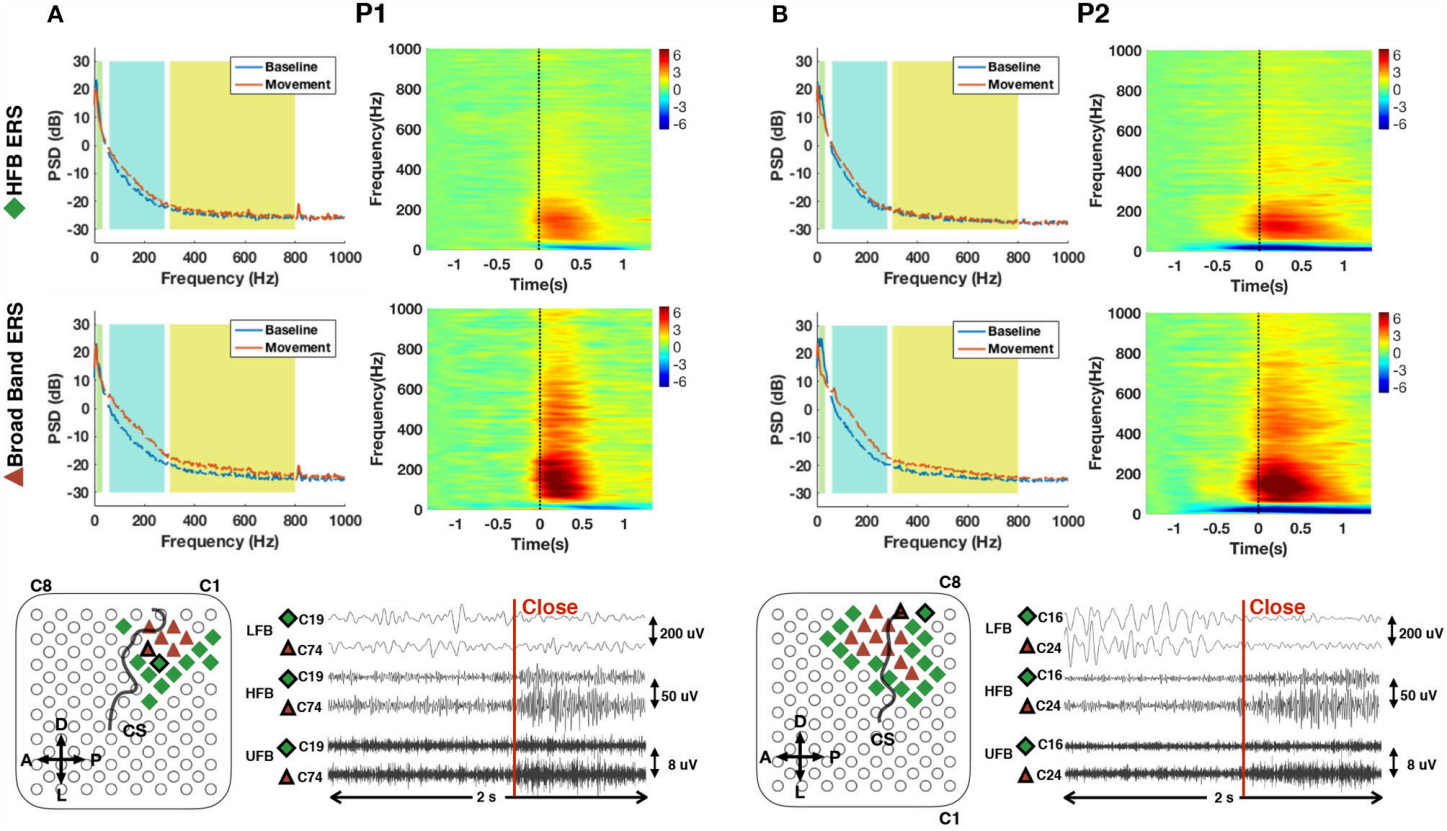
Average power spectral density (PSD) and normalized time frequency maps of channels with exclusively significant HFB ERS (upper row) and broad band ERS (middle row) for P1 **(A)** and P2 **(B)**. LFB (8–32 Hz), HFB (60–280 Hz) and UFB (300–800 Hz) ranges are shaded in different background colors in the PSD plots. All time frequency maps were displayed from 6 to −6 dB. Channels with significant HFB ERS and broad band ERS are represented by green diamond and red triangle, respectively, on the grid of the bottom row. Central sulcus (CS) was marked by a black curve. Orientations were denoted by “A” (anterior), “P” (posterior), “D” (dorsal) and “L” (lateral). An epoch of 2 s of subband filtered signal around movement onset (close) from neighboring channels with different ERS characteristics is also shown at the bottom row for P1 (C19, C74) and P2 (C16, C24). The locations of the channel pairs are highlighted on the grid. The scale of each subband is provided on the right side of the data plot.

The average power spectral density estimations and normalized time-frequency maps for channels with significant ERS in traditional high gamma band are shown in the first row of Figure 6. The compensated PSD clearly followed the 1/f nature of ECoG spectra and reached a noise floor after around 400 Hz in the baseline state. In the movement state, a visible noise floor was evident after 800 Hz. ERD covering a range including alpha and beta band (8–32 Hz) and ERS in traditional high gamma band (60–280 Hz) during hand movement can be clearly observed in the spectra and time-frequency maps of Figure 6. In addition, we observed that in a limited set of channels (i.e., 6 channels for P1 and 11 channels for P2), ERS presented itself in a broader frequency range (60–800 Hz) (second row of Figure 6). In order to differentiate the observed broad band ERS from traditional HFB-ERS, we further divided the broad band activity of 60–800 Hz into HFB and UFB. HFB was restricted within 60–280 Hz to be consistent with the high gamma frequency band modulations found in ours and others previous studies. UFB was selected to be above 300 Hz reaching up to 800 Hz. The lower bound of UFB (i.e., 300 Hz) was at the “elbow” position in the power spectrum of hand movement (red). The channel positions associated with HFB-ERS (diamond) and UFB-ERS (triangle) are visualized in the bottom row of Figure 6.

According to the average time-frequency maps in Figure 6, it was observed that LFB-ERD in P1 initialized after movement onset, whereas LFB-ERD in P2 was observed at around 500 ms before movement onset. In addition, the absolute amplitude of LFB-ERD for P1 was smaller than ERS in HFB, whereas for P2 LFB-ERD was more prominent than HFB-ERS. It is also worthwhile to mention that although the hands of the patients were kept closed for 2–3 s during the task (Figure 5), the ERS in HFB or UFB generally vanished after 1.5 s following movement onset (Figure 6).

The bottom row of Figure 6 shows raw subband filtered signals from two neighboring channels (C19, C74 for P1 and C16, C24 for P2) around hand movement onset. The corresponding channel positions were circled out in black on the grid. For P1, significant ERS in both HFB and UFB were observed in C74. However, there was only a significant ERS in HFB in the adjacent channel C19. Interestingly, for P1, ERD in LFB was weak despite clear ERS in HFB in some channels. For P2, both channels had clear ERD in LFB in addition to ERS in HFB. Similarly to P1, channel 24 had significant ERS in HFB and UFB, whereas the neighboring channel C16 had a significant ERS only in HFB. These results suggest that there was well delimited spatially localized activation in UFB.

### 3.2. Cortical localization and topographical analysis of subband modulations

**Figure 7:**
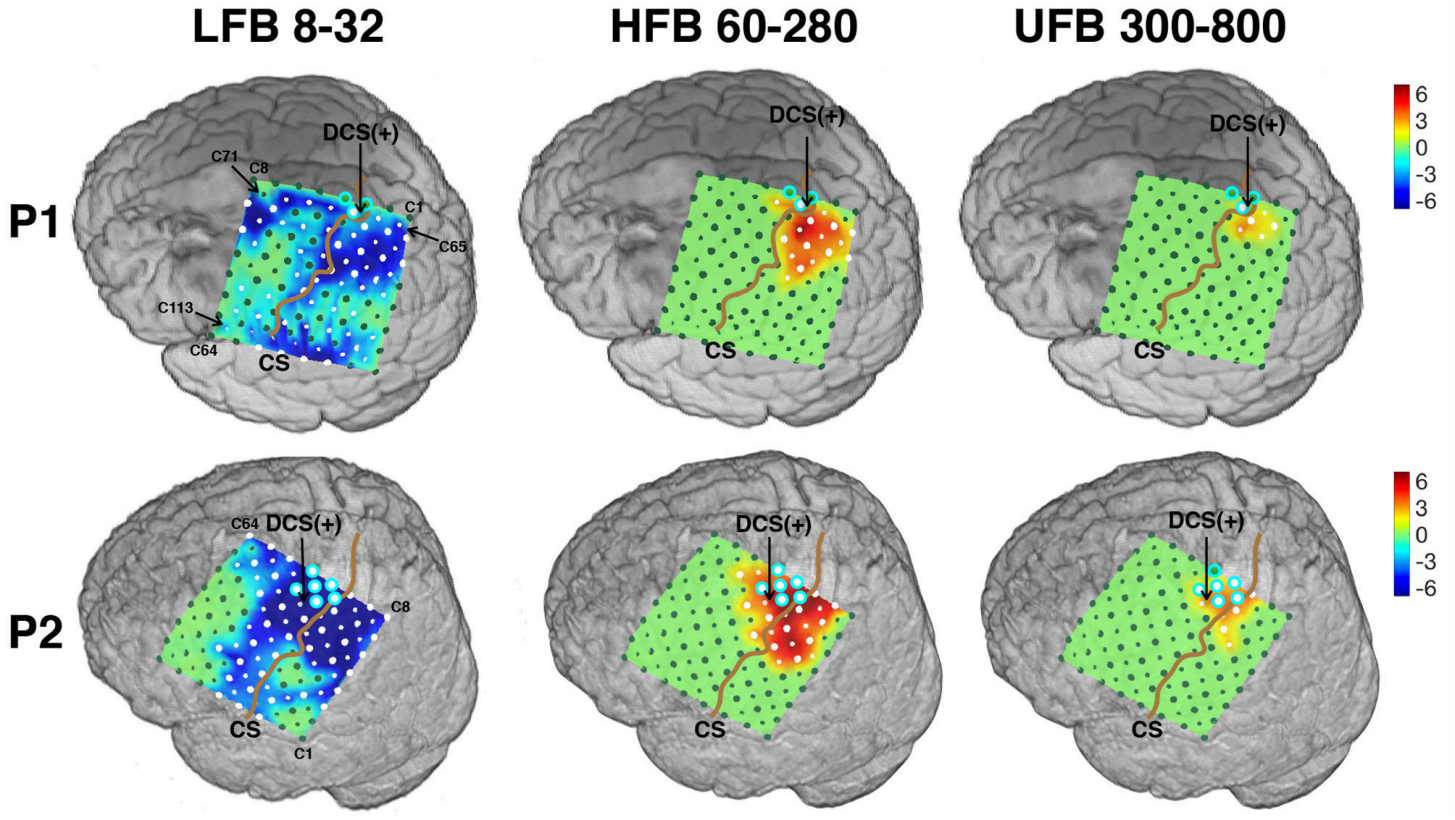
Spatial spectral activities mapped onto subject's individual 3D MRI texture rendering. All maps were thresholded to reveal only channels with significant power changes of 25% or more (one tailed *t*-test, *p* < 0.05, *fdr* < 0.05). DCS (+) site for hand function was pointed out by an arrow for each subject. Contacts located on the “hand knob” were outlined in cyan. The central sulcus (CS) was accentuated by a brown curve. Significantly activated channels were marked as white. Peak activated channels (P) were also pointed out. The naming conventions of both large and small contacts are shown in the first map. All maps were displayed from −6 to 6 dB.

The ECoG grid localization was registered on the segmented 3D volume of the brain using a CT based method for P1 and a photo based method for P2 (Figure 7). For P1, intraoperative photo and postoperative CT based registration methods show concordant results on localizing the exposed electrodes relative to the central sulcus. ECoG spectral activity were mapped onto the individual brain surface using registered electrode locations. The ECoG grids were positioned to maximize the clinical requirements and partially covered the motor cortical “hand knob” for both patients.

The spatial distribution of significant modulation of ECoG subbands are visualized on the 3D cortical mesh in Figure 7. All spatial maps are displayed with power scale from −6 to +6 dB. The ECoG electrodes marked in white color in Figure 7 represent channels with significant modulations in the respective subbands. The central sulcus is identified and accentuated by a black line on the figure. The DCS positive sites for hand function are also pointed out.

For each subband, the number of significantly activated channels anterior (*N*_*a*_) and posterior (*N*_*p*_) to the central sulcus are shown in Table 2. Compared to the traditional ERS in HFB, ERS in UFB was lower in magnitude (Figure 6B) and more focally localized in both subjects. For P1, 6 (5.3%) channels were associated with significant UFB-ERS, while there were 17 (15%) channels with significant HFB-ERS. More than half of channels (63) were associated with significant LFB-ERD. For P2, 11 (9.7%) channels were associated with significant UFB-ERS and 27 (23.9%) channels were associated with significant HFB-ERS. In addition, 66 (58.4%) channels exhibited significant LFB-ERD.

**Table 2:** Distributions of spatial patterns relative to CS and DCS(+).

**ID**	**LFB**			**HFB**			**UFB**		
	***N*_*a*_/*N*_*p*_**	***d*_*cs*_ (mm)**	***d*_*p*_/*d*_*c*_ (mm)**	***N*_*a*_/*N*_*p*_**	***d*_*cs*_ (mm)**	***d*_*p*_/*d*_*c*_ (mm)**	***N*_*a*_/*N*_*p*_**	***d*_*cs*_ (mm)**	***d*_*p*_/*d*_*c*_ (mm)**
P1	24/39	17 ± 10.6	44.3/32.9	2/15	9.5 ± 6.4	12.7/14.4	1/5	6 ± 3.2	12.7/9.0
P2	40/26	14.5 ± 8.9	19.0/11.9	13/14	8.6 ± 6.1	10.6/8.9	6/5	5.7 ± 4.1	3.5/6.8

For P1, most of channels with significant ERS in HFB (15 over 17 in total) and UFB (5 over 6 in total) were posterior to the central sulcus which is presumed to be a somatosensory area. For P1 39 channels with LFB-ERD were distributed on the posterior side while 24 channels were on the anterior side. Significant modulations in all three subbands were found in one (C67) out of three contacts located on the “hand knob.” The significant channel (C67) also appeared to be the closest to the DCS(+) site. Generally, the sensorimotor related activations in all three subbands for P1 were distributed posterior to the central sulcus, in contrast to the activations for P2.

The spatial organization of HFB and UFB ERS channels for P2 covered sensorimotor areas anterior and posterior to the central sulcus (Figure 7). Specifically, 13 out of 27 HFB-ERS channels and 6 out of 11 UFB-ERS channels were anterior to the central sulcus. For P2, 40 out of 66 channels associated with significant LFB-ERD were distributed on the anterior side. All six contacts covering the “hand knob” exhibited significant LFB-ERD and HFB-ERS while five of them exhibited significant UFB-ERS. The channel without significant UFB-ERS (C40) was at the anterior boundary of the precentral gyrus. The DCS(+) site was found slightly laterally to the anatomic “hand knob.”

**Figure 8:**
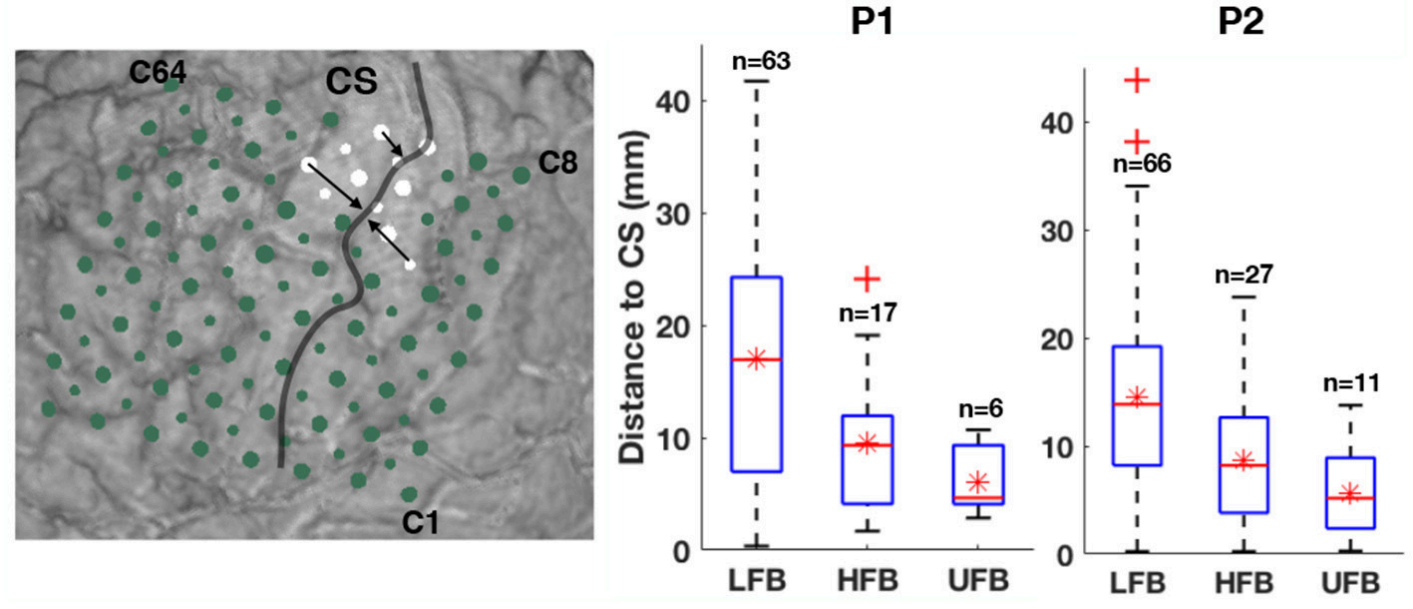
The distances of significant UFB-ERS contacts relative to the central sulcus are partially denoted by black arrows on the MRI 3D rendering of P2 (left). Box and whisker diagram of relative distances to central sulcus (CS) in millimeter within significantly modulated channels in LFB, HFB, and UFB. Sample sizes are shown above the boxes. Red line denotes the median value. Red star denotes the mean value. Outliers of trials are marked as red crosses.

The left subplot of Figure 8 illustrates the relative distance of significantly UFB-ERS contacts to the central sulcus on the MRI 3D rendering of P2. The distributions of average distances of ECoG mapping in LFB, HFB and UFB relative to the central sulcus (*d*_*cs*_) are shown in the box plots on the right side of Figure 8 and listed in detail in Table 2. The UFB modulated channels were consistently closer to the central sulcus compared to LFB and HFB modulated channels. Specifically, the average distance relative to central sulcus of significantly active UFB channels was 6 mm (±3.2 mm) for P1 and 5.7 mm (±4.1 mm) for P2. While the average distance of HFB modulated channels increased to 9.5 mm (±6.4 mm) and 8.6 mm (±6.1 mm) for P1 and P2, respectively. For both patients, the average distance of LFB modulated channels relative to central sulcus was greater than both HFB and UFB modulated channels.

The distance values of *d*_*p*_ and *d*_*c*_ of the spatial pattern of LFB, HFB, and UFB relative to DCS(+) sites are also given in Table 2. For P1, the channel with HFB-ERS peak was the same as for the UFB-ERS peak, which was recorded in C74. However, the distance between the centroid of the UFB map and the DCS(+) site (*d*_*c*_) was smaller (9.0 mm) than the distance between the centroid of the HFB map and the DCS(+) site (14.4 mm). For P2, the peak of UFB-ERS was recorded in C31 while the peak of HFB-ERS was in C85. The latter was farther from the DCS(+) site compared to C31 (*d*_*p*_: 3.5 vs. 12.7 mm). The centroid distance (*d*_*c*_) was also slightly smaller for UFB (9.5 mm) than for HFB (10.6 mm). For both patients, the LFB spatial pattern was even farther from the DCS(+) sites compared to HFB in terms of both *d*_*p*_ and *d*_*c*_. In general, both peak distance and centroid distance revealed that UFB mapping was in closer proximity with the DCS(+) sites compared to HFB (Table 2).

### 3.3. Onset timing analysis of HFB and UFB ERS

**Figure 9:**
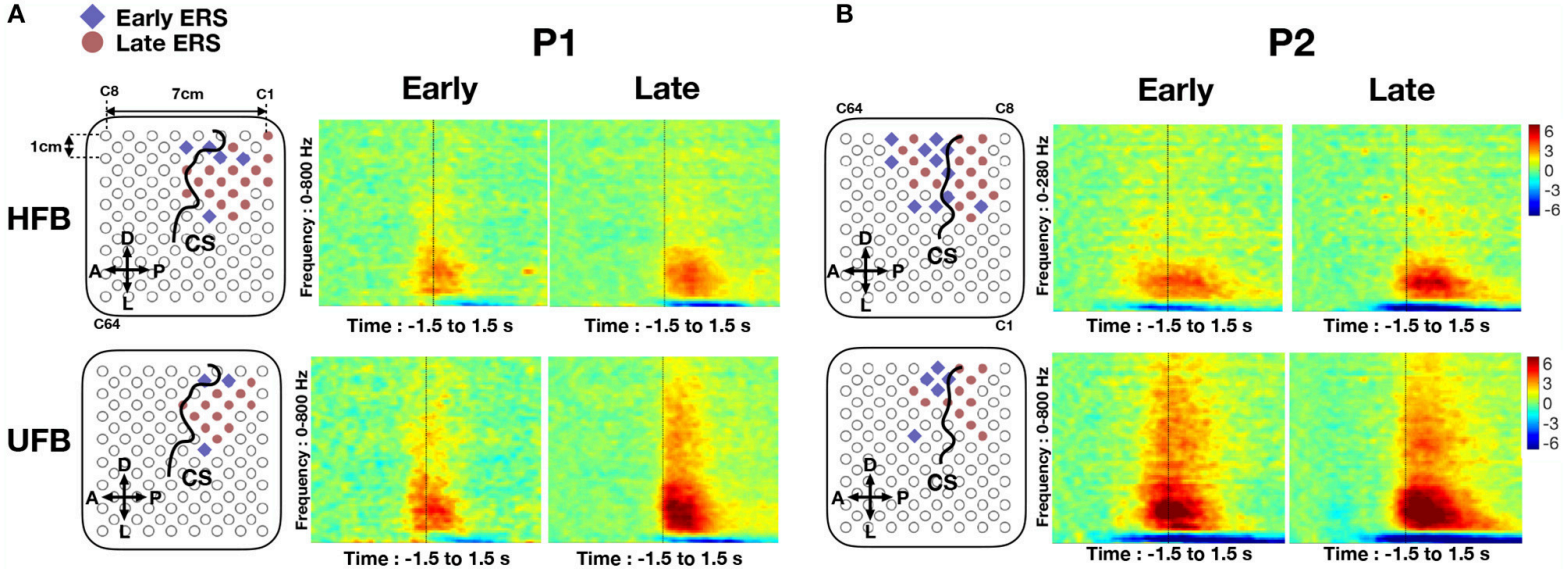
**(A)** The early-ERS (blue diamond) and late-ERS (red circle) channel distributions on the grid and average time-frequency maps in HFB (top) and UFB (bottom) frequency range for P1. **(B)** Same analysis results for P2. Non significant channels were left as blank in the grid map. Central sulcus (CS) was denoted by a black curve. Orientations were denoted by “A” (anterior), “P” (posterior), “D” (dorsal) and “L” (lateral). The average time-frequency maps were visualized in a frequency range of 0–800 Hz and covered a period of −1.5 s to 1.5 s. All time-frequency maps were displayed in −6 to 6 dB.

Channels with early HFB-ERS (blue) and late HFB-ERS (red) are visualized on the first row of Figure 9. Time-frequency maps averaged in early and late HFB-ERS groups are also shown. Channels with significant ERS in UFB were excluded while averaging the time-frequency maps of HFB-ERS channels. For P1, five channels had an early HFB-ERS and were localized adjacent to the central sulcus on both anterior (C67, C68) and posterior (C88, C10, C11) sides. The channel (C67) located on the “hand knob” with significant modulations exhibited early HFB-ERS. For P2, 10 over 11 channels that exhibited early HFB-ERS were located on or anterior to the central sulcus. Four of the early HFB-ERS were located on the “hand knob.” For both patients, most of the channels posterior to the central sulcus were associated with late HFB-ERS.

Channels with early UFB-ERS (blue) and late UFB-ERS (red) are visualized on the second row of Figure 9. Average time-frequency maps for the early and late UFB-ERS channels are shown on the right side. For P1, three channels exhibited early UFB-ERS. Specifically, C66 and C88 were posterior while C67 was anterior to the central sulcus and located on the “hand knob.” In contrast, for P2, all five channels that exhibited early UFB-ERS were located anteriorly to the central sulcus, and four of those were located on the “hand knob.” Most of the late UFB-ERS channels were posterior to the central sulcus. For both patients, early ERS in HFB and UFB generally appeared at the anterior channels of the activated region (Figure 9).

### 3.4. Small vs. large contact groups

**Figure 10:**
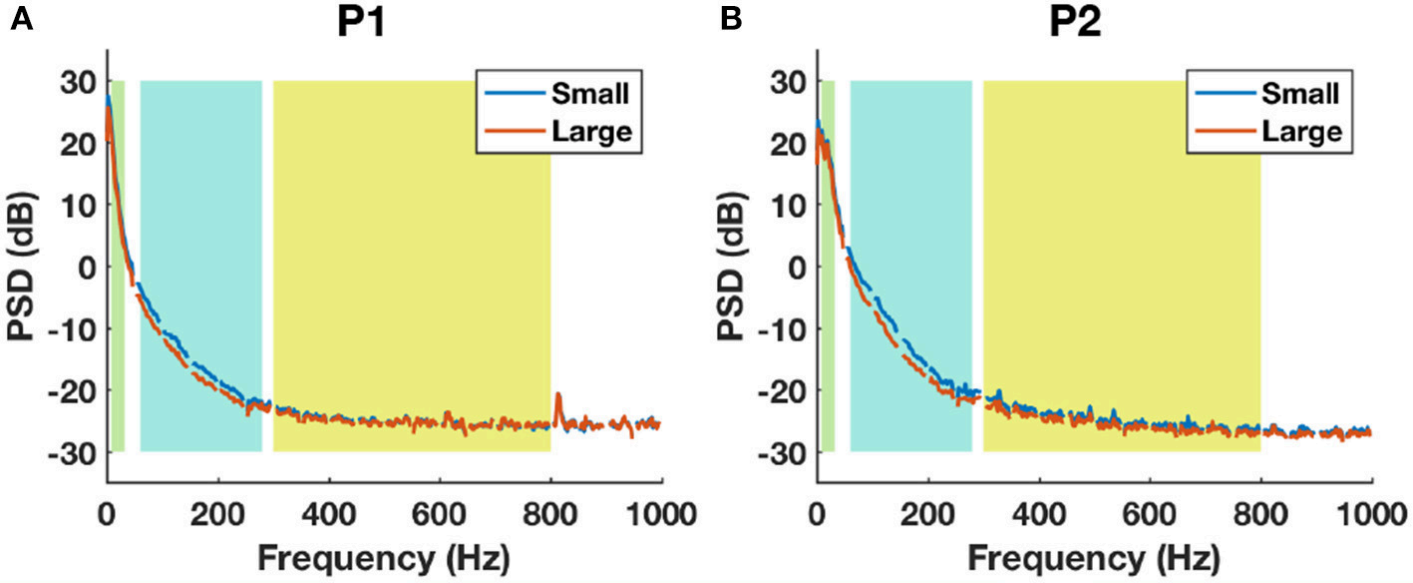
The baseline power spectral density estimations between small (blue) and large (red) contact groups using all channels in P1 **(A)** and P2 **(B)**. Shaded backgrounds from left to right indicate respectively LFB, HFB, and UFB.

The average baseline PSD plots between small and large contacts for each patient are shown in Figure 10. For both subjects, the HFB band power was higher in small contacts (blue) compared to large contacts (red). The noise floor estimated from 800-1000 Hz of the spectrum for P1 was 1.86±0.16 μ*V* for small contacts (48 channels) and 1.83±0.15 μ*V* for large contacts (45 channels). For P2, the noise floor estimated was 1.62±0.25 μ*V* for small contacts (41 channels) and 1.53±0.23 μ*V* for large contacts (55 channels). For both subjects, although the statistical tests did not yield any significant difference between the noise floor of small and large contacts (P1: *p* = 0.4, P2: *p* = 0.08), the noise level tended to be higher in the smaller contacts.

**Figure 11:**
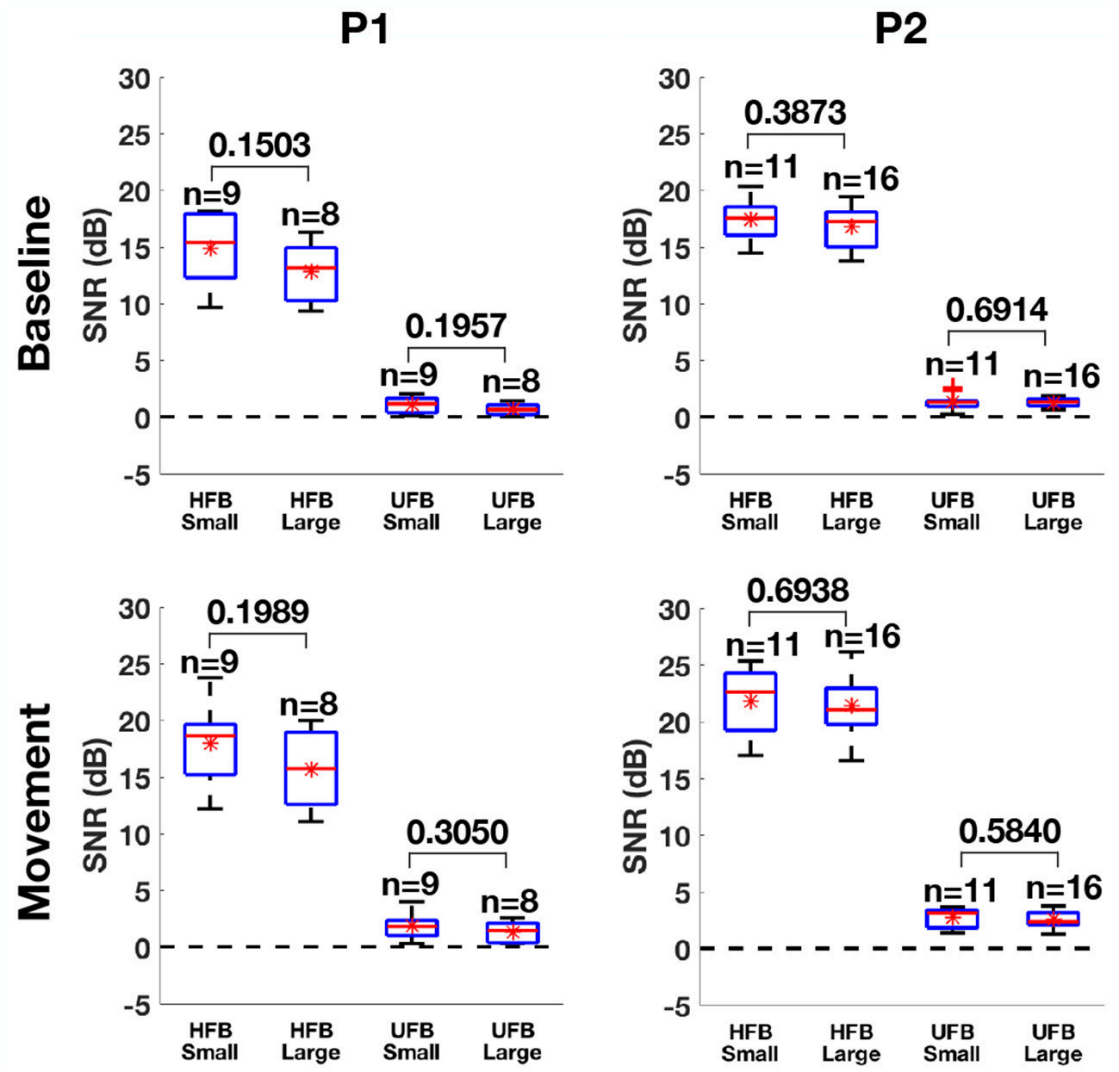
The boxplots of SNR in HFB and UFB between small and large contacts for P1 **(left)** and P2 **(right)** computed from baseline (upper) and movement (bottom). The sample number of each group is displayed above the corresponding box. The *p*-value of two-tailed Student's *t*-test between small and large contacts is also shown. Red star denotes the mean value while the red band within the box denotes the median. The noise floor (0 dB) is represented by the horizontal dash line.

The boxplot of SNR in HFB and UFB during baseline and movement between small and large contacts are shown in Figure 11. For P1, the average SNR was slightly higher in small contacts (9 channels) compared to large contacts (8 channels) in each band during both baseline (HFB: 17.4 > 16.8 dB, UFB: 1.33 > 1.25 dB) and movement (HFB: 18 > 15.7 dB, UFB: 1.86 > 1.34 dB). For P2, the average SNR was also higher in small contacts (11 channels) compared to large contacts (16 channels) during baseline (HFB: 14.9 > 12.8 dB, UFB: 1.05 > 0.65 dB) and movement (HFB: 18.5 > 17.2 dB, UFB 2.19 > 2.02 dB). However, the significance test did not yield any significant difference during both baseline (P1-HFB: *p* = 0.15, P1-UFB: *p* = 0.2, P2-HFB: *p* = 0.39, P2-UFB: *p* = 0.69) and movement (P1-HFB: *p* = 0.2, P1-UFB: *p* = 0.31, P2-HFB: *p* = 0.69, P2-UFB: *p* = 0.58).

**Figure 12:**
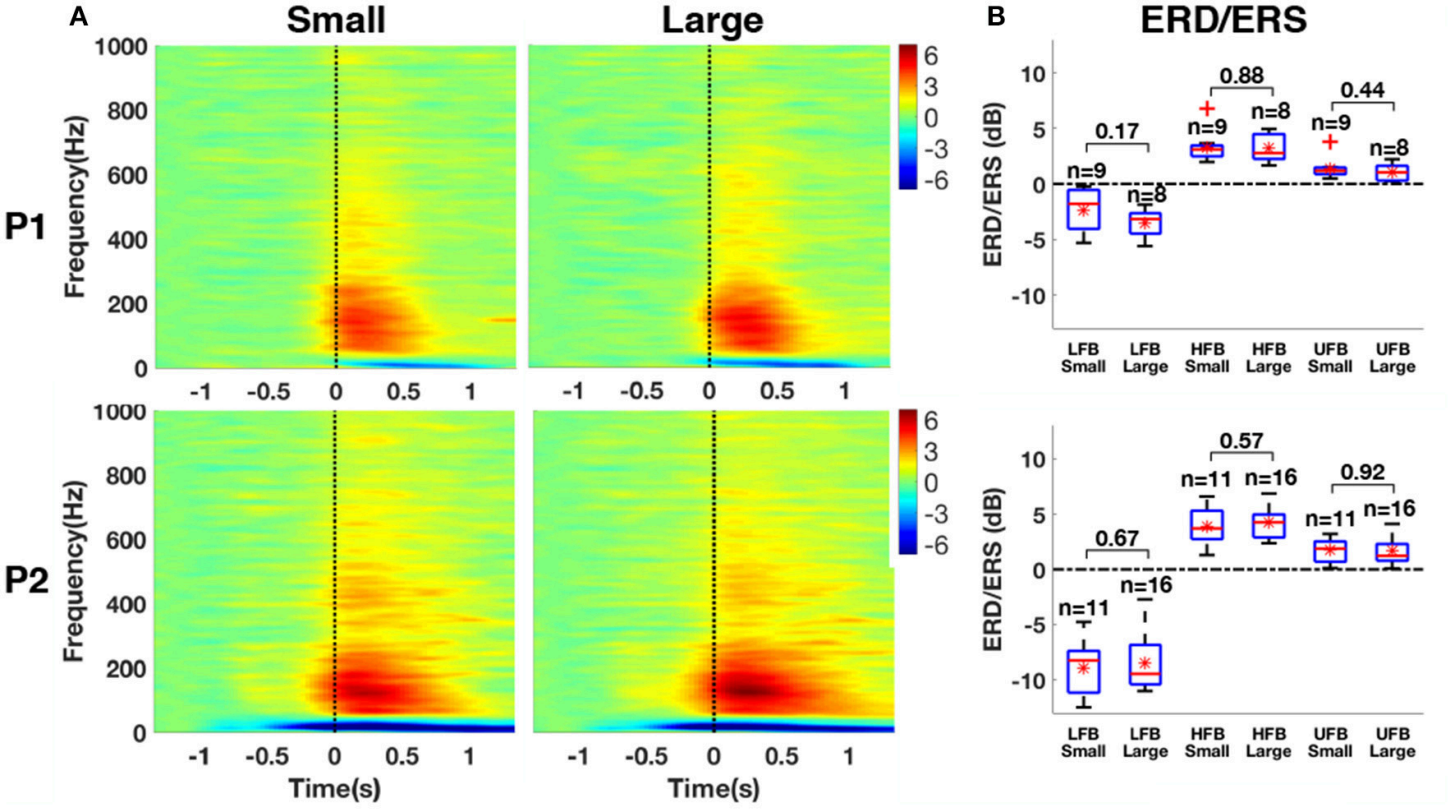
**(A)** Average time-frequency maps between small and large contact groups with significant ERS in HFB. **(B)** Box-and-whisker plots of the magnitude of ERD in LFB, ERS in HFB, and ERS in UFB between small and large contact groups. Red asterisk denotes mean value, the red band within the box denotes the median and the outliers are represented by red plus signs.

The normalized time-frequency maps averaged between small and large contact groups with significant ERS in HFB are shown for both patients in Figure 12. All groups revealed ERS (red) in HFB and ERD (blue) in LFB. The results of ERD/ERS magnitude for each subband compared between small and large contact groups are provided in Figure 12B. The statistical tests did not yield any significant difference between small and large contacts in any of the frequency bands that we investigated [LFB (P1: *p* = 0.17, P2: *p* = 0.67); HFB (P1: *p* = 0.88, P2: *p* = 0.57); UFB (P1: *p* = 0.44, P2: *p* = 0.92)].

## 4. Discussion

The frequency bands of interest in previous ECoG studies have generally been restricted at up to 250 Hz (Miller et al., 2007a,b; Leuthardt et al., 2012; Vansteensel et al., 2013). A recent study revealed that power modulations of up to 500 Hz were associated with cognitive tasks (Gaona et al., 2011). In this study, ECoG was sampled at 2.4 kHz, which enabled us to study movement related spectral modulations of up to 1.2 kHz. The most important observation in this study is that in both patients ERS in ultra-high frequency of 300-800 Hz arose from a tightly localized cortical region close to the central sulcus. Although pathological high frequency oscillations (HFO) in epilepsy patients has been found to be reaching 800 Hz (de la Prida et al., 2015), the UFB found here was task-related and time-locked to movement onset. In addition, UFB oscillations lasted for several hundred milliseconds which is well beyond the typical duration of pathological fast ripples (<0.1 s) found in epileptogenic zones (Urrestarazu et al., 2007). Furthermore, no after-discharges were observed in the significant UFB-ERS channels for both patients during the ECoG recording phase. These points support the existence of non-pathological 300–800 Hz modulation of cortical activity related to hand movements. Due to the delimited spatially localized feature of UFB modulations, the typical clinical grids with large inter-electrode spacing might fail to capture them consistently.

The average time-frequency maps in Figure 6 also revealed that both HFB and UFB modulations generally appeared at the beginning of the movement and gradually vanished after about 1.5 s even though the hand closure state was kept for 2–3 s. This correlation between ERS and the dynamic phase of movements has been observed in other ECoG studies of hand open/close movements (Jiang et al., 2017a), as well as of center-out reaching tasks (Ball et al., 2008). The spatial maps revealed that significant ERS in 300–800 Hz only occurred in a subset of electrodes with significant high gamma band ERS (60–280 Hz) (Figure 7). The distribution of UFB-ERS was closer to DCS(+) sites compared to HFB-ERS. However, since the DCS procedure was partially performed in this study due to after-discharges sometimes observed while stimulating the cortex, further studies with more comprehensive DCS procedures are required to test its correlation with UFB-ERS.

It is not yet conclusive as to which of these HFB- and UFB-ERS reflect motor activation or sensory feedback since they were localized on both sides of the central sulcus for both patients, and their modulation started either before or in coincidence with movement onset. Previous studies have shown that both primary motor cortex (M1) and somatosensory cortex (S1) were activated during attempted movements of individuals with spinal cord injury (SCI) (Cramer et al., 2005) or tetraplegia (Wang et al., 2013) as well as during motor imagery of able-bodied subjects (Porro et al., 1996; Lacourse et al., 2005). A recent ECoG BMI study also demonstrated that high decoding accuracy can be achieved on differentiating various hand gestures by using channels from S1 (Branco et al., 2017). For both patients in this study, most of the early-ERS channels were located at the anterior part of the activated region. However, the existence of early-ERS in HFB posterior to the central sulcus of P2 can be viewed as evidence of S1 activation in top-down movement preparation. There is still controversy as to whether pre-movement activations of S1 truthfully represent the top-down efference copy or merely reflect the somatosensory feedback of subtle muscle contractions before movement onset (Ryun et al., 2017).

Although the statistical test of SNR in HFB and UFB recorded by small and large contact groups during movement did not yield any significant difference, the small contacts were consistently associated with slightly higher average values in both subjects. This might indicate that small contacts has a slight advantage in detecting the high frequency rhythms compared to the large contacts. This could be due to the localized nature of high frequency rhythms of the cortical activity (Su and Ojemann, 2013). Statistical test of ERD/ERS in small and large contact groups did not yield any significance difference either. These results indicate that, in terms of ERD/ERS analysis, ECoG studies using a high-density grid with smaller contact size (Marathe and Taylor, 2013; Bleichner et al., 2014; Hotson et al., 2016; Wang et al., 2016; Jiang et al., 2017a) provide comparable information, in terms of time-frequency characteristics, as studies using standard larger clinical ECoG grids. It is also worthwhile to mention that UFB generally has much lower SNR than HFB during movements (Figure 11). This could lead to extra difficulty in detecting the UFB modulations. However, although small, the SNR of UFB oscillation in HFB-ERS channels (Figure 7) was still significantly higher than the noise level (0 dB) during both baseline and movement (one-tailed Student's *t*-test, *p* < 0.05). The oversampling process executed by the amplifier provides improved SNR in data acquisition which might have helped with the detection of ERS in UFB range. Nevertheless, besides the possibility of being generated by small cortical circuits, the localized spatial characteristic of UFB-ERS could also be due to its low SNR.

Motor functional reorganization has been extensively studied in stroke patients but less so in brain tumor patients. However, functional reorganization is more likely to occur in LGG patients than in stroke (Desmurget et al., 2007) and high-grade glioma (HGG) patients (Bryszewski et al., 2012) due to slowly growing tumor. In this study, the LFB-ERD, HFB-ERS, and UFB-ERS activation patterns for P1 were found mainly posteriorly to the central sulcus, in contrast to those for P2. Although, one channel (C67) on the “hand knob” of P1 remained active, the magnitude of its modulation was smaller compared to the posteriorly located channels. In addition, for P1 the majority of early HFB-ERS and early UFB-ERS were posterior to the central sulcus, again in contrast to those for P2. This posterior localization of motor-related activity for P1 probably results from a functional reorganization due to the combined factors of tumor progression and surgical resection (Seitz et al., 1995; Duffau, 2001; Bryszewski et al., 2012). This is consistent with other reports of postcentral shift of the hand motor function in LGG patients (Seitz et al., 1995). Consequently, we can surmise that the hand motor function area for P1 has reorganized posteriorly to the central sulcus through proliferation of novel, injury-induced corticocortical connections between the premotor and somatosensory cortex (Dancause et al., 2005). However, there was no obvious sign of functional reorganization under the brain area covered by the ECoG grid of P2. We assume that the history of previous tumor resection and of longer tumor progression for P1 since childhood elicited a greater brain functional reorganization than for P2.

Most functional reorganization studies so far were based on either fMRI (Bryszewski et al., 2012; Kurabe et al., 2016) or DCS (Duffau et al., 2002, 2003). Although deemed as the gold standard of functional mapping, DCS does not map functional motor behavior, and might induce seizures by injecting current to the cortex (Boulogne et al., 2016). On the other hand, fMRI is non-invasive and provides high spatial resolution. However, it indirectly estimates neuronal activity by measuring related hemodynamic changes and has poor temporal resolution (seconds). In comparison, ECoG can safely measure neuronal activity with high temporal resolution, whereas the spatial resolution is dependent on the density of the electrode grid. Unique spectral and temporal information related to functional activity can also be obtained from ECoG using dedicated signal processing techniques. As a result, ECoG functional mapping combined with robust electrode registration techniques could be a useful modality to complement existing techniques on both functional mapping and functional reorganizations studies. A better understanding of functional reorganization, especially in low-grade brain tumor patients, could improve the surgical outcome by maximizing the excision while preserving the reorganized functioning area.

## 5. Conclusions

In both patients, we were able to record movement-related ERD and ERS in multiple channels using a hybrid high-density ECoG grid. Consistently in both patients, ERS reached up to 800 Hz in a limited number of channels. To the best of our knowledge, this is the first time that ERS in an ultra-high frequency band up to 800 Hz of ECoG has been reported. In both patients, LFB-ERD was spatially broader compared to HFB- and UFB-ERS. We also explored the movement related patterns projected onto the individual MRI. We found that UFB-ERS observed around anatomic “hand knob” was more focally localized and resided closer to the central sulcus and DCS(+) sites than HFB-ERS. In addition, most of the sensorimotor-related cortical activation for P1 was found to be posterior to the central sulcus, in contrast to P2. This suggests a potential functional reorganization of the motor cortical functional area in P1. Finally, we did not find any significant difference between the task-related band power changes captured by the small and the large ECoG contacts.

This study has provided new understanding toward how the brain conveys information during functional hand motor tasks in terms of different frequency ranges of neural oscillatory activity. Also, we believe that the newly discovered UFB has great potential for increasing the precision of motor brain functional mapping. This unique wide band activity needs to be further explored in a larger population in our ongoing functional mapping and functional reorganization studies.

## Author contributions

NI designed the study. NI, SL, AS, AA, SK, PS, and CG collected the data. TJ and NI conducted the analysis and TJ, NI, and GP wrote the manuscript and interpreted the results. AS, AA, SK, PS, and CG performed the surgeries, contributed to data collection, imaging and DCS procedures and interpretation of the results. AS and CG performed the behavioral tests during surgery, evaluated the condition of subjects, and contributed to interpretation of the results. All authors reviewed the manuscript and approved the final version of the manuscript.

### Conflict of interest statement

The authors declare that the research was conducted in the absence of any commercial or financial relationships that could be construed as a potential conflict of interest.
